# Antifactor H Autoantibody Characterization in Atypical Hemolytic Uremic Syndrome

**DOI:** 10.1016/j.ekir.2025.08.019

**Published:** 2025-08-21

**Authors:** Vinaya Dulipati, Anu Kaskinen, A. Inkeri Lokki, Juha Kotimaa, Marcel Messing, Juuso Tainio, Anne Räisänen-Sokolowski, Timo Jahnukainen, Seppo Meri

**Affiliations:** 1 Department of Bacteriology and Immunology, Translational Immunology Research Program, University of Helsinki, Helsinki, Finland; 2 Department of Paediatric Nephrology and Transplantation, New Children’s Hospital, Helsinki University Hospital, Helsinki, Finland; 3 Heart and Lung Centre, Helsinki University Central Hospital, Helsinki, Finland; 4 VTT Technical Research Center of Finland, Espoo, Finland; 5 Department of Pathology, Helsinki University Hospital and University of Helsinki, Helsinki, Finland; 6 Diagnostic Center, Helsinki University Hospital, Helsinki, Finland

**Keywords:** aHUS, alternative pathway, autoantibody, complement system, factor H, pediatric patient

## Introduction

In atypical hemolytic uremic syndrome (aHUS), now referred to as complement-mediated thrombotic microangiopathy (c-TMA),S1 alternative pathway dysregulation is central to pathogenesis, either because of genetic defects (50%–60%) or autoantibodies against factor H (FH) in 6% to 25% (European) and up to 55% (Indian) of cases.^1^, ^2^, ^3^

FH is a 155 kDa serum protein composed of 20 complement control protein (CCP) domains, which inhibit the alternative pathway by preventing C3 activation on self-cell surfaces and in solution.^4^, ^5^, ^6^ The C-terminal, CCP19-20 of FH are involved in cell surface recognition and binding to C3b.^4^, ^5^, ^6^ In anti-FH antibody–associated aHUS, autoantibodies target the CCP19-20 region and inhibit FH-mediated regulation on endothelial cells, whereas the fluid phase regulatory effect of FH remains intact.^4^^,^^7^^,^^8^

Here, we describe a pediatric case of anti-FH associated c-TMA and FHR-1 deficiency caused by *CFHR3-1* deletion. The autoantibody, referred as kAb in this paper targeted CCP19-20 and hence inhibited FH binding to C3d and the GM3 ganglioside. Furthermore, it lowered the threshold of complement activation via the alternative pathway on a trigger surface. Despite the functional activity and persistently high levels of kAb, the patient has remained in remission after the initial aHUS episode.

## Results

**Figure 1:**
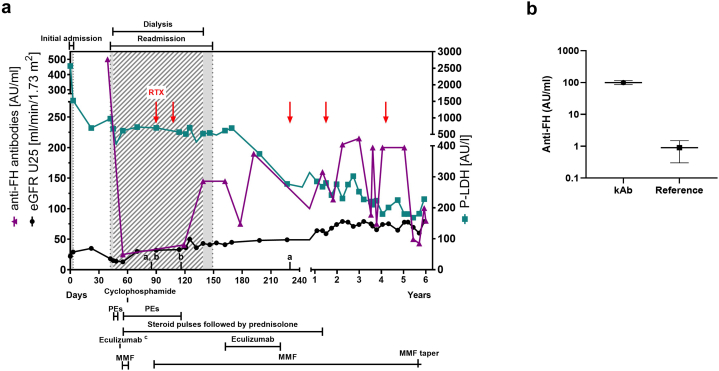
Case description and clinical parameters of kidney function, hemolysis and factor H (FH) antibodies in relation to follow-up time and treatment. (a) eGFR calculated by CKiD U25 with creatinine and cystatin C, P-LDH (values as per HUS Diagnostic Center, Helsinki, Finland) and anti-FH antibody levels (AU/ml) from the initial admission to the last follow-up. All anti-FH autoantibody values were normalized against the starting anti-FH autoantibody level set arbitrarily at 500. The hospital stay is highlighted with grey background and time on dialysis with diagonal lines. An 8-year-old, previously healthy girl presented to the emergency department after 10 days of abdominal pain, vomiting, poor appetite, mild headache, subfebrile temperatures (up to 38 °C) and upper respiratory tract infection symptoms. She had no history of bloody diarrhea. She had microangiopathic hemolytic anemia, thrombocytopenia, and acute kidney injury. Thereby she was diagnosed with HUS. She was admitted for 3 days with supportive treatment and no suspicion of c-TMA despite her repeatedly negative stool culture and PCR for EHEC. Two weeks after the discharge further complement diagnostics were performed due to continuing hemolysis (Supplementary Figure S1). Six weeks after initial presentation, she was admitted because of increased hemolysis and oliguric acute kidney injury requiring dialysis. At readmission her previously drawn complement testing revealed highly elevated levels of anti-FH antibody (a, b), and she was diagnosed with an anti-FH antibody-associated c-TMA. (b) The first anti-FH antibody level showed 100 times more anti-FH IgG binding than our laboratory standard ”high normal” serum sample from a donor with *CFHR3-1* deletion. PE was started daily but discontinued after the fourth session for a week due to gastrointestinal bleedings, whereafter PE was restarted for 7 weeks. Because of discontinuation of PE and insufficient therapeutic response ECU was started. However, administration was discontinued because anaphylactic reaction, and no further ECU was given at that point. As regards the immunosuppression, the patient was first started on methylprednisolone pulses followed by oral prednisolone taper, and MMF 1000 mg/m^2^/day. MMF was changed to cyclophosphamide after 5 days because of PRES possibly attributed to MMF, hypertension, or TMA itself. Although the autoantibody level decreased by induction therapy, hemolysis continued and the patient remained dependent on dialysis. Thus, 6 weeks after initiation of aHUS treatment a kidney biopsy was performed to assess disease severity and activity versus chronicity. The biopsy revealed active TMA (Supplementary Figure S1). RTX was started instead of continuing cyclophosphamide, and MMF was restarted. Three months after the admission, the patient was weaned off dialysis and discharged with MMF, tapering prednisolone, 4 antihypertensive agents, furosemide, alfacalcidol, and sulfadiazine/trimethoprim prophylaxis for *Pneumocystis* prevention. However, because of continuing hemolysis and partly rebounded autoantibody levels ECU was restarted for 2 months after which hemolysis decreased and repeated kidney biopsy showed rather a chronic than active TMA. The patient has received additional RTX 3 times, once because of partly rebounded autoantibody levels and prophylactically when ECU and steroids were discontinued after 2 months and 14 months, respectively. MMF tapering was initiated 5.6 years after the initial presentation. ECU, eculizumab; eGFR, estimated glomerular filtration rate; aHUS, atypical hemolytic uremic syndrome; MMF, mycophenolate mofetil; PE, plasma exchange; P-LDH, plasma lactate hydrogenase; PRES, posterior reversible encephalopathy syndrome; RTX, rituximab; TMA, thrombotic microangiopathy; ^a^Kidney biopsy; ^b^A positive blood culture for ESBL *Escherichia coli* with concomitant fever of 38.3 °C on day 85 but otherwise clinically stable. On day 116, the patient developed pericardial effusion requiring pericardial drainage and fever, but cultures remained negative. Both infections were treated with i.v. antibiotics. Whether these infections really served as precipitating factors remains unknown. ^c^Prior to the first ECU dose the patient received meningococcal (ACWY and B), pneumococcal and *Haemophilus influenzae* vaccinations and ciprofloxacin prophylaxis for 2 weeks was started.

**Figure 2:**
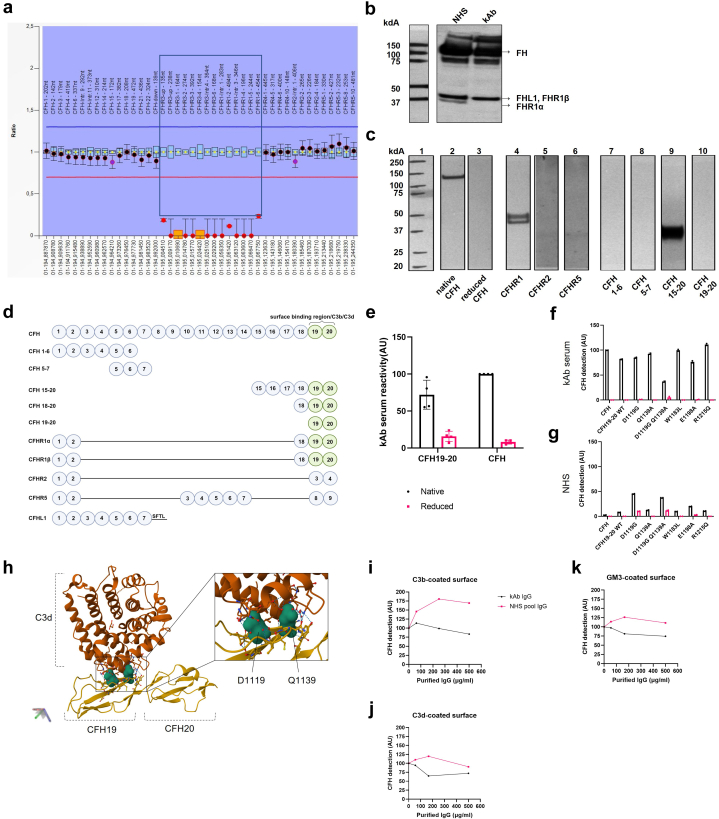
Characteristics of the FH antibody, *CFHR3-1* deletion and evaluation of kAb binding to FH and its fragments. (a) Characteristics of the anti-FH antibody and *CFHR3-1* deletion. MLPA showed *CFHR3-1* deletion in patient DNA. The normal detection ratio is 1 (between blue and red horizontal lines). The sequence probes between TTAGTCCGAGGT-AGAAAGGGACAT upstream of *CFHR3* and ATCAATCATAAA-ATGCACACCTTT located in exon 6 of *CFHR1* (inside the box) were not detected at all (orange box markers) or were detected by a residual signal close to zero. (b) Immunoblotting with a polyclonal anti-FH antibody was used to detect FH, FHL1, FHR1α, and FHR1β in serum samples from a control NHS and the patient serum. The control serum sample showed bands of FH at 155 kDa, FHL1 at 43kDa, FHR1β at 42kDa, and FHR1α at 37 kDa. Because of similar apparent molecular weights and migration in the gel, the FHL-1 and FHR1β bands are overlapping. Supporting the MLPA, the blot showed that patient serum was FHR1α absent. (c). Western blot analysis of the patient serum binding to different FH antigens. Lane 1 is the molecular weight standard. Lanes 2 to 10 show the binding of kAb to FH and FH fragments. Lane 2 shows binding of the patient autoantibody to a distinct band presenting the native (nonreduced) form of FH. When FH was reduced before loading to the gel, no binding was seen (lane 3). The autoantibody bound to purified human native FHR1α and FHR1β (lane 4); however, no binding was observed to recombinant FHR2 (lane 5) or FHR5 (lane 6) proteins. Recombinant, nonreduced FH products containing domains 1 to 6 (lane 7) or 5 to 7 (lane 8) did not show autoantibody binding. In contrast with the other FH fragments the nonreduced 40kDa FH15-20 construct bound the autoantibody very strongly (lane 9). No binding of kAb to the recombinant FH19-20 fragment was seen by immunoblotting (lane 10), possibly because of a conformational alteration of domain 19 in SDS-PAGE electrophoresis. However, clear binding was seen in an ELISA test (Figure 2e). (d) Mapping of the target epitopes of the anti-FH antibody kAb. Sequence alignment of the tested CCPs suggest the FH19-20 region as the likely autoantibody target. (e) To determine the specific kAb binding site, we assessed the autoantibody reactivity to recombinant native FH19-20 and its reduced form together with native and reduced FH. Recombinant FH19-20 fragments and purified human FH (both 10 μg/ml; native and reduced) were coated on a microtitre plate wells and allowed to react with kAb followed by anti-human IgG detection. Binding to the reduced proteins was minimal, which is in line with the observations in Figure 2c. Data from 2 biological replicates each having 2 technical replicates and normalized against the OD450 readout of kAb reactivity to native FH. The error bars represent SEM. The recombinant mutants (native and reduced) were coated on a microtiter plate and overlaid with (f) kAb serum or (g) NHS as a control followed by anti-human IgG detection. The patient autoantibody bound approximately equally well to most of the FH19-20 purified native single amino acid mutant fragments D1119G, T1184R, K1186A, E1198A, R1203A, R1206A, R1210A, R1215Q, and purified human FH. Decreased binding of kAb to the D1119G/Q1139A double mutant suggests its significance in preserving the tertiary structure of the autoantibody binding site and that the kAb binding site is located in domain 19 of FH. Data from 2 technical replicates. The error bars represent SD. (h) Protein structure of FH19-20 (with residues D1119 and Q1139) and C3d in complex previously solved by Morgan *et al.*S7 was visualized using RCSB Protein Data Bank Mol∗ (WebGL) with the code 3OXU. D1119 and Q1139 are closely located residues that bulge out from the CCP domain 19 of FH. Next, binding of biotinylated FH binding was tested in an ELISA set-up. Different amounts of total IgG from patient serum (kAb) and NHS were preincubated in solution with purified biotinylated human FH (6 μg/ml) to assess the FH binding to purified (i) human C3b (5 μg/ml), (j) C3d (5 μg/ml), or (k) GM3 (5 μg/ml) coated wells. Representative data from 3 biological replicates. kAb inhibited FH binding to C3b in a slightly dose-dependent manner, whereas NHS IgG showed increased FH binding to C3b. In comparison, a somewhat stronger inhibition of FH binding was observed by kAb on C3d-coated surfaces and GM3-coated surfaces. Probably the autoantibodies bound FH in solution during the preincubation step and prevented the interaction of FH domains 19 and 20 with the surface associated targets. Inhibition of binding to GM3 could be due to steric hindrance. ELISA, enzyme-linked immunosorbent assay; FH, anti-factor H; MLPA, multiplex ligation-dependent probe amplification.

The patient case is described with clinical parameters and management in detail in Figure 1a. In summary, a previously healthy 8-year-old Caucasian girl was diagnosed with HUS and 6 weeks after initial presentation confirmed with an anti-FH associated c-TMA. The multiplex ligation-dependent probe amplification analysis of the patient’s DNA showed a homozygous deletion of *CFHR3-1*S2 (Figure 2a); however, the HUS gene panel with 9 genesS3 was negative (data not shown). The patient was planned for treatment with plasma exchanges (PEs), methylprednisolone pulses followed by oral prednisolone taper, and mycophenolate mofetil (MMF).^3^^,^^9^ However, her management became complex because of treatment-related complications and insufficient response (Figure 1a). Altogether, 22 PEs were performed over 9.5 weeks, and in addition to the initial plan she received 1 dose of eculizumab (ECU) and was on cyclophosphamide for 1 month after which it was switched to rituximab. Eventually, she was weaned off dialysis and discharged after 3 months. However, because of continuing hemolysis and increasing autoantibody levels, ECU was restarted for 2 months. Since then, the patient has remained in remission with varying anti-FH autoantibody levels. She has received 3 additional rituximab infusions and was started on MMF taper 5.6 years after initial presentation. At 6 years from initial presentation, the patient continued to thrive with enalapril treatment and an estimated glomerular filtration rate of 79 ml/min per 1.73 m^2^; no proteinuria (19 mg/mmol creatinine). In addition, the patient was recently diagnosed with autoimmune thyroiditis.

In characterizing the kAb (Supplementary Methods), we found anti-FH reactivity in different IgG subclasses, indicating polyclonality of the autoantibody (Supplementary Figure S2), but dominance for the IgG3 subclass^1^^,^^7^^,^S4^,^S5 and signals for both kappa and lambda light chains, but stronger for lambdaS4 (Supplementary Figure S2). kAb lowered the threshold for complement activation via the alternative pathway on a trigger (*Salmonella* LPS) surface as observed by formation of C5b-9 in the presence of 2% NHS (Supplementary Figure S3).

When testing the target epitope of the patient autoantibody (Supplementary Methods), kAb bound to native FH, FHR1, and FH15-20 (Figure 2c). Domain sequence alignment and direct binding analysis showed FH domains 19 or 20 as the autoantibody binding regionS5 (Figure 2d and e). Importantly, kAb bound only to native but not to reduced FH or FH19-20 fragments (Figure 2e). Therefore, the binding of the kAb autoantibody depends on 1 or more conformational epitopes located on FH19-20. Furthermore, the native forms of the purified FH19-20 mutants containing various amino acid mutations^7^ bound more strongly to the IgG from the serum samples than the reduced forms (Figure 2f and g). Interestingly, a weaker binding, and similar to the level of the NHS IgG control, was seen with the native double mutant D1119G Q1139A (Figure 2f and g). Both residues D1119 and Q1139 are located adjacent to each another on domain 19 (Figure 2h).^7^^,^S6^,^S7 This result indicates that the kAb binding site is in FH domain 19.

Separate binding sites for the C3d region of C3b are located both on domain FH 19 and 20.^7^ In addition, domain 20 has been shown to bind especially to the GM3 ganglioside.S8 Our studies (Figure 2i–k) showed that kAb impaired FH binding to C3b, C3d, and GM3.

## Discussion

Our study characterized the anti-FH autoantibody binding site and functions of an autoantibody from a pediatric patient with anti-FH–associated c-TMA, who, despite the persistently elevated antibody levels, has remained in remission after the treatment initial aHUS episode and while on MMF.

Previous studies have reported the dominant epitope of the anti-FH antibody–associated HUS to be in the C-terminus of FH,^7^^,^^8^^,^S5^,^S6^,^S9–S12 mirroring our results. To our knowledge, our study is the first demonstration of a conformationally sensitive binding epitope in FH domain 19 in the C-terminus of FH in anti-FH antibody–associated aHUS. Importantly, the kAb autoantibody bound only native FH and FH19-20 fragments, indicating that the autoantibody binding site is conformationally sensitive, losing its structural integrity, when reduced because of the loss of disulfide linkages. Our study shows the amino acid residues, D1119 and Q1139 on FH CCP19 may contribute to the binding because a joint mutation in these 2 residues reduced kAb binding. Mutation D1119G has previously been linked to genetically determined aHUSS13 and Q1139A has been shown to influence FH binding to C3d.^7^

Past studies have shown that anti-FH autoantibody impaired FH binding to C3b and glomerular endothelial cells in aHUS.S9^,^S14^,^S15 We observed that kAb influenced FH binding to purified human C3b, C3d, and GM3 pointing toward the function of kAb in impairing FH binding and possibly self-cell surface recognition, which may promote complement attack against such surfaces. The homozygous deletion of *CFHR3-1* is known to predispose to aHUSS16^,^S17; and FHR1 deficiency associated with anti-FH autoantibody formation in aHUS has been explained previously with an induced neoepitope model,S18 but the mechanisms leading to the production of anti-FH autoantibodies are not clear.S9^,^S14

Owing to the autoimmune nature of anti-FH antibody–associated c-TMA, the treatment strategy consisted of antibody removal by PE and immunosuppression to inhibit further production of antibodies.^2^^,^^3^^,^^9^ However, since ECU became available in 2009, it has been more commonly used for the treatment of anti-FH antibody–associated HUS, and there is increasing evidence that ECU and immunosuppression without PE could be safely used as a first-line treatment even in children with severe kidney damage.^9^^,^S19–S21 In our patient, antibody levels decreased by PEs, followed by improvement in kidney function; however, hemolysis resolved only after rituximab and ECU regimen for 2 months. Nevertheless, delayed treatment initiation and meandering management because of various treatment-related complications may have restrained her response to the treatment.S22 Due to associations between increased antibody levels and relapse, MMF treatment for a minimum 2 years is recommended to minimize the risk for relapses^9^^,^S20^,^S23 However, autoantibodies can persist in remission in up to 88% of patients as in our patient^1^^,^S23 Our patient has remained in remission; however, because of the fluctuating antibody levels, she received rituximab and was not started on MMF tapering until 5.6 years after her initial presentation.

Our study presents a thorough analysis of a case of anti-FH antibody–associated c-TMA in a pediatric patient, the treatment interventions, and its challenges across 6 years of disease monitoring. Furthermore, we explored the characteristics of the anti-FH autoantibody, which may have influenced how the disease manifested. Altogether, the results contribute to our understanding of the function of the anti-FH autoantibodies in c-TMA and disease progression.

## Disclosure

The authors declare no conflict of interest.

## Supplementary Material

Supplementary File (PDF) **Supplementary Methods. Supplementary References. Figure S1.** Findings of the first kidney biopsy. **Figure S2.** kAb IgG subclass ELISA results show polyclonality of kAb. **Figure S3.** ELISA analysis of C5b-9 formation by complement activation via the alternative pathway by *Salmonella* LPS in the presence of pooled NHS and protein G purified total IgG from patient and that from NHS pool.
