# Supplementary material for: Antifactor H Autoantibody Characterization in Atypical Hemolytic Uremic Syndrome
Source: Kidney Int Rep. 2025 Aug 21;10(11):4081–6. doi: 10.1016/j.ekir.2025.08.019 (PMC12639820; doi:10.1016/j.ekir.2025.08.019)
Supplement: Supplementary File (PDF) — Supplementary Methods. Supplementary References. Figure S1. Findings of the first kidney biopsy. Figure S2. kAb IgG subclass ELISA results show polyclonality of kAb. Figure S3. ELISA analysis of C5b-9 formation by complement activation via the alternative pathway by Salmonella LPS in the presence of pooled NHS and protein G purified total IgG from patient and that from NHS pool. [file mmc1.pdf]

Supplementary Materials

Supplementary Methods

We used genetic analysis and functional protein assays to characterize the disease mechanism in the index patient.

Genetic characterisation

Complement gene investigations were performed by exome sequence and copy number variation analyses (Hemolytic Uremic Syndrome Panel (*ADAMTS13, C3, CD46, CFB, FH, FHR5, CFI, DGKE, THBD*), Blueprint Genetics Oy, Helsinki, Finland). Complement component levels (C3, C4) were determined by nephelometry on Siemens BN ProSpec (Siemens Healthcare, Erlangen, Germany) and the complement pathway activities by Wieslab® complement system screen kit (Svar Life Science, Malmö, Sweden) at HUS Diagnostic Center, Division of Clinical Microbiology, Helsinki, Finland.

Copy number variations within the *CFH/CFHR* gene region in chromosome 1 were assessed by multiplex ligation-dependent probe amplification (MLPA) using the SALSA MLPA P236 *CFH* region probemix applied as described per the manufacturer's (MRC Holland) protocols to the patient’s DNA extracted from peripheral blood. Samples from a group of donors with known genotypes were used to interpret the results and as technical controls.

Factor H protein studies

Purified CFH was obtained from Complement Technology (Texas, USA). Cloning, expression, and purification of recombinant wild-type CFH19–20 and mutant CFH19-20 proteins having 14 single-point mutations have been previously described(**7; S6; S27**). Recombinant CFH fragments CFH1-6,

CFH5-7 and CFH15-20 were produced as described**(S18; S28; S29)**. CFHR-1 was a kind gift from Dr Mihály Józsi and was purified from whole plasma of healthy individuals as described(**S30**). Biotinylation was carried out using EZ-LinkTM NHS-Biotin N-Hydroxysuccinimidobiotin (Thermo Fisher Scientific, Finland) per the manufacturer's instructions. The purities of proteins were confirmed by sodium dodecyl sulfate polyacrylamide gel electrophoresis (SDS-PAGE), and the concentrations were determined with Qubit 4 fluorometer (Thermo Fisher Scientific, Finland). CFH was detected on SDS-PAGE and immunoblotting was used for the detection of CFH and CFHR-proteins as well as for the detection of anti-CFH antibodies in the patient serum. Purified CFH (Complement Technology, USA), CFH fragments or serum samples (1:100) were run on SDS-PAGE (4-12% Bis-Tris gradient) gels (Thermo Fisher Scientific, Finland). For detection of CFH/CFHRs, goat anti-human factor H polyclonal antibody (CalBioChem, Germany) followed by HRP-conjugated polyclonal rabbit anti-goat IgG (DAKO Cytomation, USA) were used.

Complement-preserved human serum (NHS) pools used in the study were prepared with serum from healthy research laboratory staff donors composed of equal numbers of female and male donors. The serum pool was aliquoted and stored at -80˚C prior to use. Protein G columns (Cytiva Sweden AB) were used to purify immunoglobulins from patient plasma, plasmapheresis samples and from NHS pool samples per manufacturer's instructions.

kAb-antibody characterization

The anti-CFH detection ELISA adapted from the Helsinki method (**S31**) was performed to assess the levels of anti-CFH autoantibody titer from patient samples. The microtiter plate wells were coated with 10μg/ml purified CFH (Complement Technology, USA). Phosphate buffered-saline containing 0.05% Tween 20 (PBS-T) was used for blocking. Patient serum was diluted 31.5 times in PBS-T and incubated. Peroxidase AffiniPure™ goat anti-human IgG (H+L) (Jackson ImmunoResearch, USA)

was used for detection, and the reaction was revealed with o-phenylenediamine dihydrochloride (OPD) and stopped with 0.5M sulphuric acid. The reaction was read at 492nm.

To determine the IgG subclass of the kAb autoantibody the abovementioned protocol was modified as follows. Coating was carried out with 2µg/ml purified CFH. Blocking was carried out with 2% NFDM. Serial dilutions of purified IgG from serum samples were incubated in the plate before detection with secondary antibodies (mouse anti-human IgG1 Fc fragment, Merck Germany; rabbit anti-human IgG2 recombinant secondary, Invitrogen Finland; mouse anti-human IgG3 hinge, Invitrogen Finland; mouse anti-human IgG4, Bio-Rad USA). Detection was carried out with Peroxidase AffiniPure™ goat anti-rabbit IgG (H+L) (Jackson ImmunoResearch, USA), Peroxidase AffiniPure™ rabbit anti-mouse IgG (H+L) (Jackson ImmunoResearch, USA), goat anti-human IgG (Lambda light chain/HRP (Invitrogen, Finland) or goat anti-human Kappa/HRP (Invitrogen, Finland). Peroxidase AffiniPure™ goat anti-human IgG (H+L) (Jackson ImmunoResearch, USA) was used as a positive control. The bound IgG was revealed using TMB, the reaction stopped with 0.5M sulfuric acid and the absorbance was read at 450nm.

The interference of kAb on CFH binding to purified human complement C3b (Complement Technology, USA), C3d (Complement Technology, USA), and GM3 (Avanti Lipids, USA) was determined similarly. Wells were coated with either 5µg/ml purified GM3 ganglioside (Avanti Lipids, USA), 5µg/ml purified human complement C3b (Complement Technology) or 5µg/ml purified human complement C3d (Complement Technology, USA) in PBS. Blocking was performed with 1% w/v bovine serum albumin (BSA). Serial twofold dilutions of total purified IgG starting from 500µg/ml in Veronal buffered saline (pH 7.3) were preincubated with 6µg/ml biotinylated purified CFH for 30 mins prior to adding to the plate. Detection was carried out with 0.5µg/ml Pierce™ Streptavidin Poly-HRP (Thermo Scientific, Finland).

The formation of C5b-9 in the presence of kAb resulting from the alternative pathway activity triggered with *Salmonella* enteritidis lipopolysaccharide S-form (LPS, Hycult Biotech, Netherlands) was measured. Wells were coated with 1µg/ml LPS in PBS containing 10mM $MgCl_2$. To measure baseline C-activation, reciprocal dilutions of NHS pool samples prepared in Tris-buffered saline (TBS; 10mM Tris base and 150mM NaCl, pH 7.4) containing 5mM $MgCl_2$ and 10mM EGTA (MgEGTA), 1% w/v BSA, and 0.05% Tween-20 (TBT/MgEGTA) were incubated in the plate. Protein G purified patient plasmapheresis samples or NHS pool were supplemented to serially diluted NHS pool samples. Biotinylated anti-human SC5b-9 (neoantigen) (Quidel Corporation, USA) 1% w/v BSA in TBS (B-TBS) was incubated followed by the reaction reveal.

Ethical statement

Informed consent was obtained from the patient and the caregivers before the study commenced. The study conforms to the principles of the Declaration of Helsinki and has been approved by the Research Ethics Committee of Helsinki and Uusimaa Hospital District (HUS/180/2020, HUS/564/2024).

Data analysis

The sequenced amplified fragments were analyzed using the Coffalyser software (MRC Holland). Microsoft Excel 2010 was used for ELISA standard curve analysis and data normalization. GraphPad Prism 9 was used to plot the graphs.

Dulipati et al., Figure S1

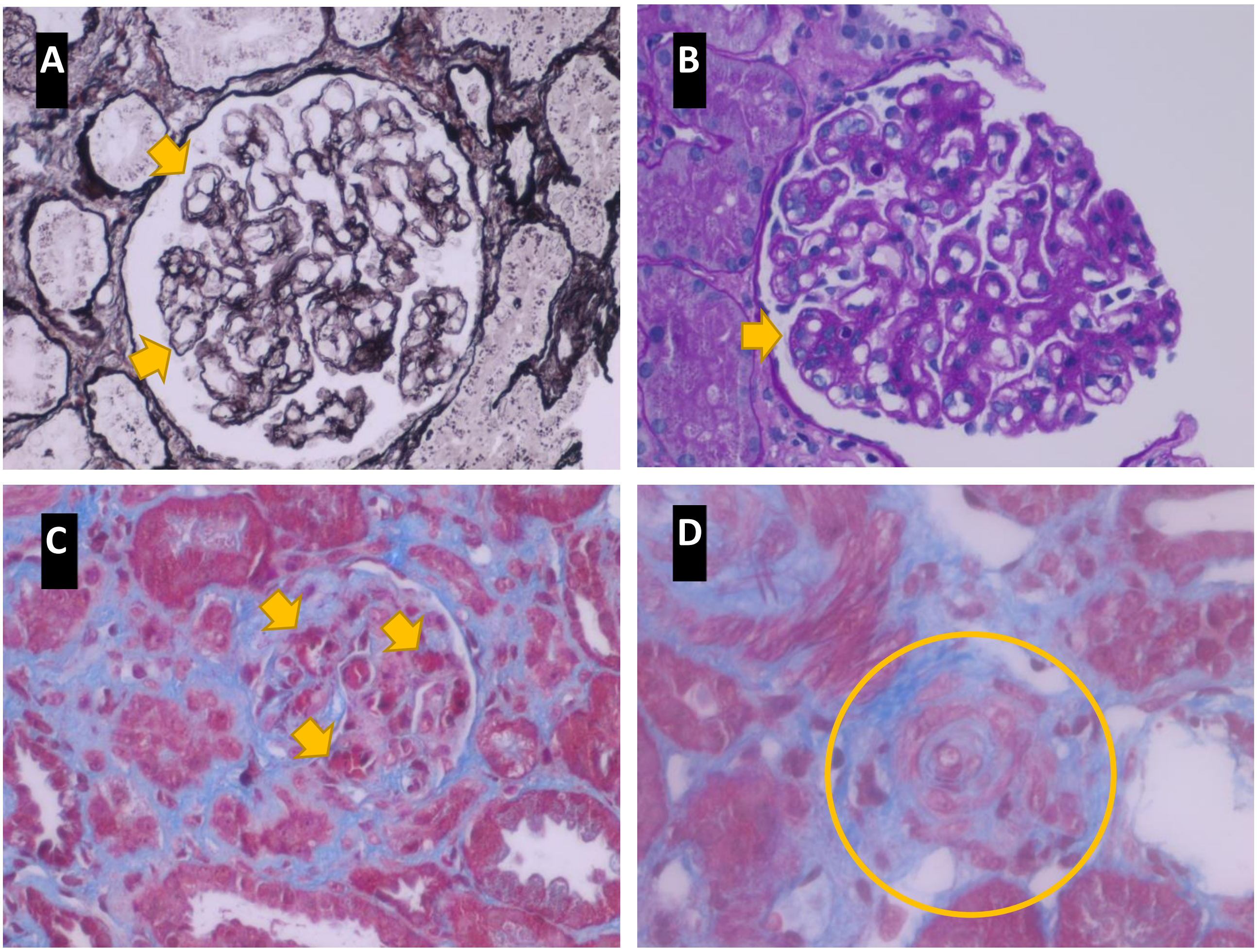

Figure S1. Findings of the first kidney biopsy. (A) The glomerular basement membranes are thickened, uneven, wrinkled and duplicated (yellow arrow) in many capillary loops (Silver methenamine, 400x). **(B)** Thick basement membranes (yellow arrow) can be seen throughout the glomeruli, but the surrounding tubulointerstitial tissue is better preserved (Periodic-Acid-Schiff, 400x). **(C)** Some of the glomeruli are occluded with microthrombi (yellow arrow, Masson Trichrome, 400x). **(D)** Some capillaries are completely occluded due to ongoing endothelial cell injury (yellow circle, Masson Trichrome, 630x).

Dulipati et al., Figure S2

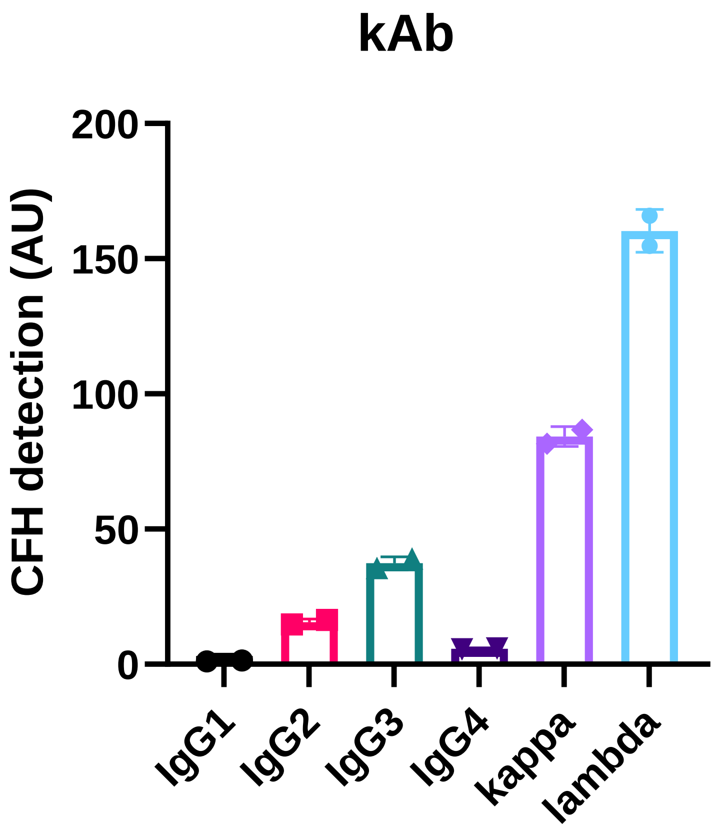

Figure S2. kAb IgG subclass ELISA results show polyclonality of kAb. Anti-FH ELISA for IgG subtyping with protein G purified patient IgG sample showed elevated IgG3 subclass-type antibody, IgG2 subclass-type antibody, and lambda light chains almost 2x that of kappa, pointing towards the polyclonality of kAb, but preference for IgG3 lambda. The data show technical replicates. Error bars represent SD.

Dulipati et al., Figure S3

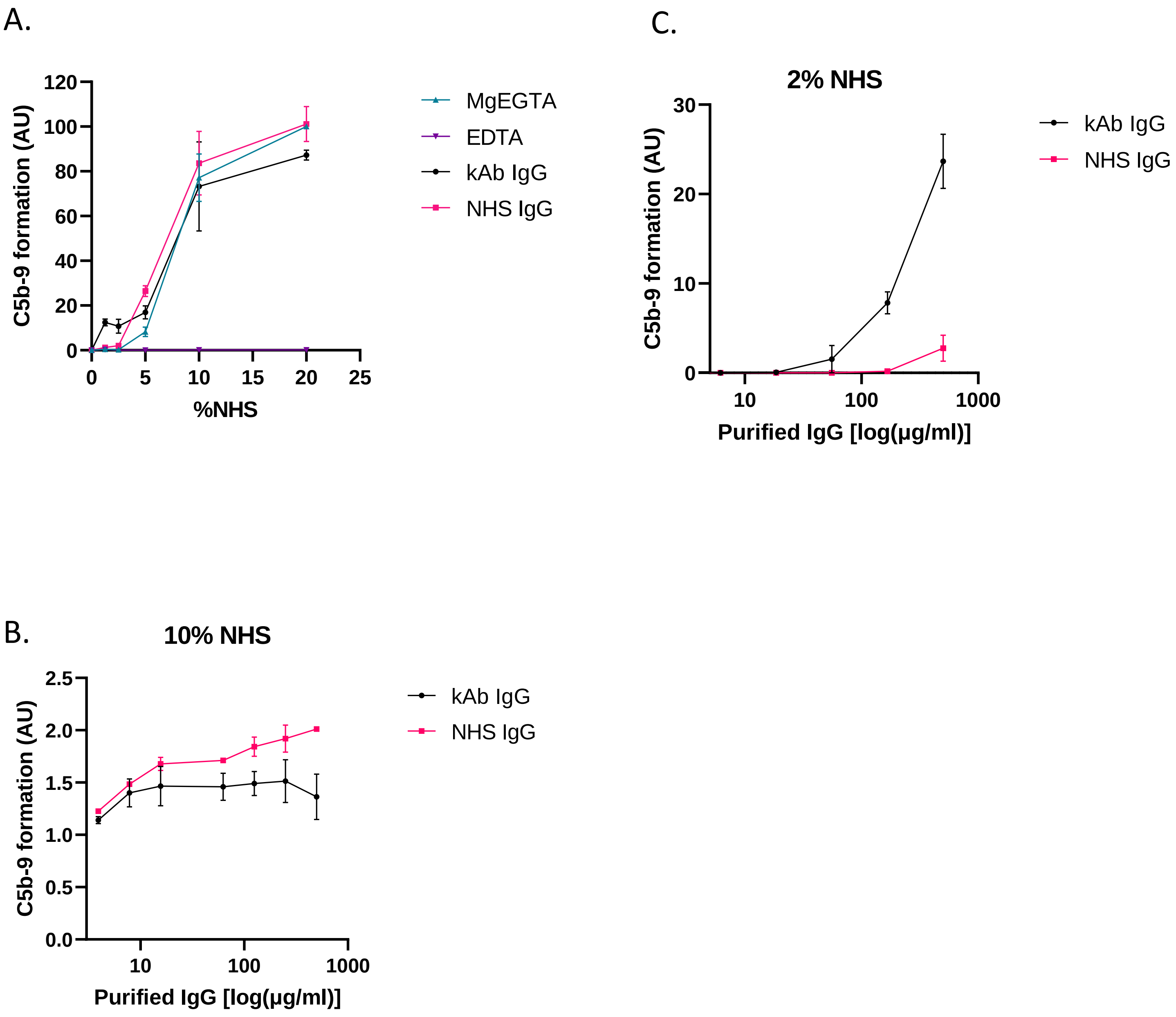

Figure S3. ELISA analysis of C5b-9 formation by complement activation via the alternative pathway by *Salmonella* LPS in the presence of pooled NHS and protein G purified total IgG from patient and that from NHS pool. Patients with anti-FH antibodies often require a trigger for TMA to manifest. At initial presentation our patient had URTI in her family and in herself, symptoms potentially attributable to a microbial infection despite no confirmed pathogen. Furthermore, her infections during the admission may have served as a precipitating factors to keep her aHUS active for a longer period as also previously reported in literature by Loirat C et al(S24). We assessed the effect of kAb on the activation of complement via the alternative pathway triggered on *Salmonella* smooth lipopolysaccharide-coated microtiter plate surfaces adapted from Fredrikson GN et al(S25). There was an increased complement C5b-9 formation in the presence of kAb **(A)** even at NHS concentration as low as 2% NHS at which the alternative pathway activity is known to be limited **(A and C)**. **(A)** C5b-9 formation was detected in reciprocal dilutions of NHS incubated in EDTA, MgEGTA, in the presence of 500 µg/ml IgG from patient, and 500 µg/ml IgG from NHS pool. Data was normalised against the OD450 value from C5b-9 formation with 20% NHS/MgEGTA. **(B)** C5b-9 formation with 10% NHS pool mixed with reciprocal dilutions of IgG from patient and from NHS IgG. Data was normalised against the OD450 value of formed C5b-9 with 10% NHS/MgEGTA taken as 1 AU. Here, the formation of complement C5b-9 in the presence of kAb was about 5 times higher than in the control. **(C)** C5b-9 formation with 2% NHS pool mixed with reciprocal dilutions of IgG from patient and that from NHS IgG. It has been reported by Seelen et al. (S26), that the activity of the alternative pathway is not clearly detected on a complement function ELISA at NHS concentrations lower than 5%, which corroborates with our observations. Notably, in 2% NHS pool the presence of 2000 µg/ml of patient IgG resulted in a signal 10 times as strong as in the control strongly indicating the role of kAb in lowering the threshold for complement activation resulting in C5b-9 formation via the alternative pathway. The persistence of high levels of the anti-FH autoantibodies could thus impair the regulatory activity of FH on the surfaces of endothelial cells and blood cells in the aHUS patient. Data was normalised against the OD450 value of formed C5b-9 with 2% NHS/MgEGTA taken as 1 AU. Representative data from two of four biological replicates.

Supplementary references

S1 Nester CM, Feldman DL, Burwick R, Cataland S, Chaturvedi S, Cook HT, Cuker A, Dixon BP, Fakhouri F, Hingorani SR, Java A, van de Kar NCAJ, Kavanagh D, Leung N, Licht C, Noris M, O'Shaughnessy MM, Parikh S V., Peyandi F, Remuzzi G, Smith RJH, Sperati CJ, Waldman M, Walker P & Vivarelli M (2024) An expert discussion on the atypical hemolytic uremic syndrome nomenclature—identifying a road map to precision: a report of a National Kidney Foundation Working Group. *Kidney Int* **106**, 326–336.

S2 Noris M, Bresin E, Mele C & Remuzzi G (2021) Genetic Atypical Hemolytic-Uremic Syndrome. *GeneReviews®*.

S3 Blueprint Genetics (2023) Hemolytic Uremic Syndrome Panel.

S4 Nozal P, Bernabéu-Herrero ME, Uzonyi B, Szilágyi Á, Hyvärinen S, Prohászka Z, Jokiranta TS, Sánchez-Corral P, López-Trascasa M & Józsi M (2016) Heterogeneity but individual constancy of epitopes, isotypes and avidity of factor H autoantibodies in atypical hemolytic uremic syndrome. *Mol Immunol* **70**, 47–55.

S5 Guo W yi, Song D, Liu X rong, Chen Z, Xiao H jie, Ding J, Sun S zhen, Liu H yan, Wang S xia, Yu F & Zhao M hui (2019) Immunological features and functional analysis of anti-CFH autoantibodies in patients with atypical hemolytic uremic syndrome. *Pediatric Nephrology* **34**, 269–281.

S6 Jokiranta TS, Jaakola VP, Lehtinen MJ, Pärepalo M, Meri S & Goldman A (2006) Structure of complement factor H carboxyl-terminus reveals molecular basis of atypical haemolytic uremic syndrome. *EMBO J* **25**, 1784.

S7 Morgan HP, Schmidt CQ, Guariento M, Blaum BS, Gillespie D, Herbert AP, Kavanagh D, Mertens HDT, Svergun DI, Johansson CM, Uhrín D, Barlow PN & Hannan JP (2011) Structural basis for engagement by complement factor H of C3b on a self surface. *Nat Struct Mol Biol* **18**, 463.

S8 Blaum BS, Frank M, Walker RC, Neu U & Stehle T (2016) Complement Factor H and Simian Virus 40 bind the GM1 ganglioside in distinct conformations. *Glycobiology* **26**, 532–539.

S9 Trojnár E, Józsi M, Uray K, Csuka D, Szilágyi Á, Milosevic D, Stojanovic VD, Spasojevic B, Rusai K, Müller T, Arbeiter K, Kelen K, Szabó AJ, Reusz GS, Hyvärinen S, Jokiranta TS & Prohászka Z (2017) Analysis of linear antibody epitopes on factor H and CFHR1 using sera of patients with autoimmune atypical hemolytic uremic syndrome. *Front Immunol* **8**, 255691.

S10 Blanc C, Togarsimalemath SK, Chauvet S, Le Quintrec M, Moulin B, Buchler M, Jokiranta TS, Roumenina LT, Fremeaux-Bacchi V & Dragon-Durey M-A (2015) Anti-factor H autoantibodies in C3 glomerulopathies and in atypical hemolytic uremic syndrome: one target, two diseases. *J Immunol* **194**, 5129–5138.

S11 Kajander T, Lehtinen MJ, Hyvärinen S, Bhattacharjee A, Leung E, Isenman DE, Meri S, Goldman A & Jokiranta TS (2011) Dual interaction of factor H with C3d and glycosaminoglycans in host-nonhost discrimination by complement. *Proc Natl Acad Sci U S A* **108**, 2897–2902.

S12 Dragon-Durey MA, Blanc C, Garnier A, Hofer J, Sethi SK & Zimmerhackl LB (2010) Anti-factor H autoantibody - Associated hemolytic uremic syndrome: Review of literature of the autoimmune form of HUS. *Semin Thromb Hemost* **36**, 633–640.

S13 Martín Merinero H, Zhang Y, Arjona E, del Angel G, Goodfellow R, Gomez-Rubio E, Ji RR, Michelena M, Smith RJH & Rodríguez de Córdoba S (2021) Functional characterization of 105 factor H variants associated with aHUS: lessons for variant classification. *Blood* **138**, 2185–2201.

S14 Song D, Liu X rong, Chen Z, Xiao H jie, Ding J, Sun S zhen, Liu H yan, Guo W yi, Wang S xia, Yu F & Zhao M hui (2017) The clinical and laboratory features of Chinese Han anti-factor H autoantibody-associated hemolytic uremic syndrome. *Pediatric Nephrology* **32**, 811–822.

S15 Józsi M, Strobel S, Dahse HM, Liu WS, Hoyer PF, Oppermann M, Skerka C & Zipfel PF (2007) Anti–factor H autoantibodies block C-terminal recognition function of factor H in hemolytic uremic syndrome. *Blood* **110**, 1516–1518.

S16 Zipfel PF, Edey M, Heinen S, Józsi M, Richter H, Misselwitz J, Hoppe B, Routledge D, Strain L, Hughes AE, Goodship JA, Licht C, Goodship THJ & Skerka C (2007) Deletion of Complement Factor H–Related Genes CFHR1 and CFHR3 Is Associated with Atypical Hemolytic Uremic Syndrome. *PLoS Genet* **3**, 0387–0392.

S17 Heinen S, Sanchez-Corral P, Jackson MS, Strain L, Goodship JA, Kemp EJ, Skerka C, Jokiranta TS, Meyers K, Wagner E, Robitaille P, Esparza-Gordillo J, Rodriguez de Cordoba S, Zipfel PF & Goodship THJ (2006) De novo gene conversion in the RCA gene cluster (1q32) causes mutations in complement factor H associated with atypical hemolytic uremic syndrome. *Hum Mutat* **27**, 292–293.

S18 Bhattacharjee A, Reuter S, Trojnár E, Kolodziejczyk R, Hyvärinen HSS, Uzonyi B, Szilágyi Á, Prohászka Z, Goldman A, Józsi M & Jokiranta TS (2015) The Major Autoantibody Epitope on Factor H in Atypical Hemolytic Uremic Syndrome Is Structurally Different from Its Homologous Site in Factor H-related Protein 1, Supporting a Novel Model for Induction of Autoimmunity in This Disease. *Journal of Biological Chemistry* **290**, 9500–9510.

S19 Ferri M, Zotta F, Donadelli R, Dossier C, Duneton C, El-Sissy C, Fremeau-Bacchi V, Kwon T, Quadri L, Pasini A, Sellier-Leclerc AL, Vivarelli M & Hogan J (2024) Anti-CFH-associated hemolytic uremic syndrome: do we still need plasma exchange? *Pediatric Nephrology* **39**.

S20 Coccia PA, Alconcher LF, Ferraris V, Lucarelli LI, Grillo MA, Arias MA, Saurit M, Ratto VM, dos Santos C & Sánchez-Luceros A (2024) Eculizumab as first-line treatment for patients with severe presentation of complement factor H antibody–mediated hemolytic uremic syndrome. *Pediatric Nephrology* **40**.

S21 de Souza RM, Correa BHM, Melo PHM, Pousa PA, de Mendonça TSC, Rodrigues LGC & Simões e Silva AC (2023) The treatment of atypical hemolytic uremic syndrome with eculizumab in pediatric patients: a systematic review. *Pediatr Nephrol* **38**, 61–75.

S22 Walle J Vande, Delmas Y, Ardissino G, Wang J, Kincaid JF & Haller H (2017) Improved renal recovery in patients with atypical hemolytic uremic syndrome following rapid initiation of eculizumab treatment. *J Nephrol* **30**, 127–134.

S23 Puraswani M, Khandelwal P, Saini H, Saini S, Gurjar BS, Sinha A, Shende RP, Maiti TK, Singh AK, Kanga U, Ali U, Agarwal I, Anand K, Prasad N, Rajendran P, Sinha R, Vasudevan A, Saxena A, Agarwal S, Hari P, Sahu A, Rath S & Bagga A (2019) Clinical and immunological profile of anti-factor h antibody associated atypical hemolytic uremic syndrome: A nationwide database. *Front Immunol* **10**, 456024.

S24 Loirat C, Noris M & Fremeaux-Bacchi V (2008) Complement and the atypical hemolytic uremic syndrome in children. *Pediatr Nephrol* **23**, 1957.

S25 Fredrikson GN, Truedsson L & Sjöholm AG (1993) New procedure for the detection of complement deficiency by ELISA. Analysis of activation pathways and circumvention of rheumatoid factor influence. *J Immunol Methods* **166**, 263–270.

S26 Seelen MA, Roos A, Wieslander J, Mollnes TE, Sjöholm AG, Wurzner R, Loos M, Tedesco F, Sim RB, Garred P, Alexopoulos E, Turner MW & Daha MR (2005) Functional analysis of the classical, alternative, and MBL pathways of the complement system: standardization and validation of a simple ELISA. *J Immunol Methods* **296**, 187–198.

S27 Lehtinen MJ, Rops AL, Isenman DE, van der Vlag J & Jokiranta TS (2009) Mutations of Factor H Impair Regulation of Surface-bound C3b by Three Mechanisms in Atypical Hemolytic Uremic Syndrome. *Journal of Biological Chemistry* **284**, 15650–15658.

S28 Hebecker M & Józsi M (2012) Factor H-related Protein 4 Activates Complement by Serving as a Platform for the Assembly of Alternative Pathway C3 Convertase via Its Interaction with C3b Protein. *Journal of Biological Chemistry* **287**, 19528–19536.

S29 Castiblanco-Valencia MM, Fraga TR, Silva LB Da, Monaris D, Abreu PAE, Strobel S, Józsi M, Isaac L & Barbosa AS (2012) Leptospiral Immunoglobulin-like Proteins Interact With Human Complement Regulators Factor H, FHL-1, FHR-1, and C4BP. *J Infect Dis* **205**, 995–1004.

S30 Kopp A, Strobel S, Tortajada A, Rodríguez de Córdoba S, Sánchez-Corral P, Prohászka Z, López-Trascasa M & Józsi M (2012) Atypical Hemolytic Uremic Syndrome-Associated Variants and Autoantibodies Impair Binding of Factor H and Factor H-Related Protein 1 to Pentraxin 3. *The Journal of Immunology* **189**, 1858–1867.

S31 Watson R, Lindner S, Bordereau P, Hunze EM, Tak F, Ngo S, Zipfel PF, Skerka C, Dragon-Durey MA & Marchbank KJ (2014) Standardisation of the factor H autoantibody assay. *Immunobiology* **219**, 9–16.
